# The influence of organizational context on the use of research by nurses in Canadian pediatric hospitals

**DOI:** 10.1186/1472-6963-13-351

**Published:** 2013-09-14

**Authors:** Janet E Squires, Carole A Estabrooks, Shannon D Scott, Greta G Cummings, Leslie Hayduk, Sung Hyun Kang, Bonnie Stevens

**Affiliations:** 1 Clinical Epidemiology Program, Ottawa Hospital Research Institute, Ottawa, Canada; 2 School of Nursing, Faculty of Health Sciences, University of Ottawa, Ottawa, Canada; 3 Faculty of Nursing, University of Alberta, Alberta, Canada; 4 Department of Sociology, Faculty of Arts, University of Alberta, Alberta, Canada; 5 The Hospital for Sick Children, Toronto, Canada; 6 Faculty of Nursing, University of Toronto, Toronto, Canada

## Abstract

**Background:**

Organizational context is recognized as an important influence on the successful implementation of research by healthcare professionals. However, there is relatively little empirical evidence to support this widely held view.

**Methods:**

The objective of this study was to identify dimensions of organizational context and individual (nurse) characteristics that influence pediatric nurses’ self-reported use of research. Data on research use, individual, and contextual variables were collected from registered nurses (N = 735) working on 32 medical, surgical and critical care units in eight Canadian pediatric hospitals using an online survey. We used Generalized Estimating Equation modeling to account for the correlated structure of the data and to identify which contextual dimensions and individual characteristics predict two kinds of self-reported research use: instrumental (direct) and conceptual (indirect).

**Results:**

Significant predictors of instrumental research use included: at the individual level; belief suspension-implement, research use in the past, and at the hospital unit (context) level; culture, and the proportion on nurses possessing a baccalaureate degree or higher. Significant predictors of conceptual research use included: at the individual nurse level; belief suspension-implement, problem solving ability, use of research in the past, and at the hospital unit (context) level; leadership, culture, evaluation, formal interactions, informal interactions, organizational slack-space, and unit specialty.

**Conclusions:**

Hospitals, by focusing attention on modifiable elements of unit context may positively influence nurses’ reported use of research. This influence of context may extend to the adoption of best practices in general and other innovative or quality interventions.

## Background

Pediatrics is not immune to the well-documented challenges of successful implementation of quality improvement initiatives, moving research evidence into practice and other knowledge translation efforts. Many reasons have been cited for the gap between the research evidence and what occurs in clinical practice including lack of research studies in child health to inform practice [1], healthcare professionals’ knowledge of the evidence, and how contextual factors impend or facilitate the implementation of research [2]. Yet, in the conventional pediatric health literature, the overwhelming focus in the implementation of research is on patient and provider outcomes with little mention of how the practice environment (or context) shapes the process of implementation [3]. One notable exception is in the pediatric social service literature where there is evidence that organizational ‘social’ context (defined as climate and culture) of the service system shapes the nature of the services provided by the professionals working in those systems [3-5]. Yet, there are many examples in the pediatric health literature of research-based implementations focused on specific clinical conditions such as asthma and pain management, occurring in multiple health care environments, with no assessment of contextual factors. In these situations typically provider and patient outcomes [6-9] are the focus. This research approach implicitly suggests that context is a constant or exerts little influence on implementation success; however, a rapidly emerging body of literature suggests otherwise and stresses the powerful influence of context [10,11].

Organizational context is now recognized as an important influence on the successful implementation of, or failure to adopt, quality improvement initiatives, research evidence, and best practices [12-14]. Recent syntheses have led to the development of theory describing the relationships among contextual factors that lead to successful implementation of innovation and quality improvement generally [15-18]. Less understood are: (1) which contextual factors influence different kinds of research use; (2) what level of context (e.g., hospital facility or unit) is most relevant to successful adoption of research; and, (3) the interacting influences that may be at play between different organizational (e.g., unit vs. hospital) levels. Microsystems theory adds a useful perspective [19-24]. This theory describes organizations as comprised of macrosystems comprised of mesosytems (i.e., centres and programs), which, in turn, consist of interrelated microsystems or clinical units. Clinical units are the ‘ground zero’ of patient care delivery, the essential building blocks of organizations [19-24]. As a result, strategies focused on the clinical unit have potential to enhance the adoption of research and ultimately to transform healthcare systems [22]. The microsystem literature acknowledges the influence of unit context [19] on outcomes and patient experiences with growing evidence that the unit is the interface at which quality patient outcomes are best achieved [22,25].

In this paper, we address the gap in knowledge related to the influence of contextual factors on nurses’ use of research evidence in pediatric settings. Notably, we build on our prior pilot study [26] where we found that a more positive perception of a unit’s leadership, culture, and evaluation was associated with higher self-reported use of research findings in practice [26]. Research use is a multidimensional construct that consists of three kinds of research use: instrumental, conceptual, and symbolic (or persuasive) [27-29]. Instrumental research use is a direct use of research knowledge. It refers to the concrete application of research in clinical practice, either in making specific decisions or as knowledge to guide specific interventions related to patient care. Conceptual research use is an indirect application of research; it refers to the cognitive use of research where the research findings may change one's opinion or mind set about a specific practice area but not necessarily one's particular action. Finally, symbolic (or persuasive) research utilization is the use of research knowledge as a political tool in order to influence policies and decisions or to legitimate a position [27-29]. The purpose of the study reported here is to identify dimensions of organizational context and individual (nurse) characteristics that influence pediatric nurses’ self-reported use of research. Specifically, we focus on instrumental and conceptual use of research.

## Methods

### Design and sample, and data collection

The study reported in this paper was conducted as part of the Translating Research on Pain in Children (TROPIC) project within the Canadian Institutes for Health Research (CIHR) Team in Children’s Pain research program. We used a cross-sectional (survey) design. Of the 15 children hospitals in Canada at the start of the study, eight met our requirements of having four or more distinct units, excluding psychiatric and emergency units, with 30 or more beds. Psychiatric and emergency units were ineligible because of the potential for adverse psychologic responses to pain and/or the low incidence of painful procedures on these units [30]. The eight eligible sites were all urban-based university-affiliated pediatric hospitals; all eight sites agreed to participate. Study units within each site were eligible for inclusion if they had a distinct geographic location and administrative structure; admitted children for periods longer than 24 hours; administered painful procedures to inpatients; and had pharmacologic, physical and psychologic interventions available for managing pain [30]. For sites with more than four eligible units, four units were randomly selected to participate to include at least one medical, one surgical and one critical care unit [30].

Data were collected for the current study at two levels: (1) clinical unit, and (2) individual healthcare professionals. Some unit (e.g., average length of patient stay) data were collected using the TROPIC unit profile survey, which was completed electronically by a research nurse at each participating hospital. Individual data, some later aggregated to the unit level, came from staff (survey) and patients (chart abstraction). Healthcare professionals (nurses, allied, practice specialists, physicians) and unit managers that met the study inclusion criteria (Additional file 1) and who could be contacted were invited to complete the TROPIC staff survey on two occasions: Time 1 (May – August 2008) and Time 2 (April – August 2011). The same units were sampled on both time points but individuals completing the survey were not linked; preventing us from being able to combine data or compare respondents across time. The survey was completed online with participant responses compiled in a centralized database. A research nurse was present on the included units on a selection of all shifts (day, evening, night) for the full data collection period (3 months) to answer questions about the study and provide eligible participants with a survey package containing a letter introducing the study, and a business card providing a Uniform Resource Locator (URL) and unique password to access the survey on-line. In this paper we report analyses that used the most current data -- staff survey (administered to nurses) and unit profile survey collected at Time 2; return of the surveys implied consent to participate.

### Ethics

Ethical approvals for this study were obtained from the Health Research Ethics Boards of the appropriate Canadian universities (application Pro00003308), as well as, the hospital ethics review boards (where applicable) for all hospitals participating in the study. All research was in compliance with the Helsinki Declaration (http://www.wma.net/en/30publications/10policies/b3/index.html).

### Study variables

#### Dependent variables

The dependent variables assessed in this study were instrumental research use (IRU) and conceptual research use (CRU). They are both included in the staff survey. IRU refers to a direct and concrete use of research evidence in practice (e.g., use of guidelines and protocols) [31]. CRU refers to the cognitive use of research where the research findings may change one's opinion or mind set about a specific practice area but not necessarily one's particular action [28,31]. Both variables were measured using single items scored on five-point frequency scales from ‘10% or less of the time' to 'almost 100% of the time’. A recent systematic review [32] indicates that these measures have been used previously to obtain reliable and valid assessments of research utilization from nurses.

#### Explanatory variables

Our explanatory variables are listed in Tables 1 and 2. We selected these variables based on those available to us in the unit profile and staff surveys. Each variable was assigned to individual- or unit-level measurement (Table 3) in a series of team meetings. One variable, unit specialty (medicine, surgery, or critical care), was assigned as part of our sampling frame to ensure equal representation across specialties. Details on all explanatory variables, including definitions, measurement, and reliability, is presented in Additional file 2.

**Table 1:** Characteristics of nurse respondents for outcome and categorical covariates (N = 735)

**Variables**		**N (%)**	**IRU**	**CRU**
			**Mean (SD)**	**Mean (SD)**
Age	20-29 years	250 (34.1)	3.831 (1.04)	3.620 (1.15)
	30-39 years	198 (27.0)	3.657 (1.11)	3.465 (1.18)
	40-49 years	151 (20.6)	3.313 (1.22)	3.424 (1.20)
	50-59 years	120 (16.3)	3.425 (1.31)	3.500 (1.26)
	≥60 years	15 (2.0)	2.800 (1.32)	3.467 (1.13)
Sex	Male	42 (5.7)	3.452 (1.04)	3.119 (1.37)
	Female	692 (94.1)	3.600 (1.18)	3.542 (1.17)
Education	Diploma/Certificate	212 (28.9)	3.175 (1.27)	3.292 (1.25)
	Bachelor	496 (67.7)	3.769 (1.07)	3.619 (1.15)
	Master or higher	25 (3.4)	3.520 (1.19)	3.440 (1.12)
Employment status	Full-Time	450 (61.3)	3.690 (1.10)	3.636 (1.13)
	Part-Time	254 (34.6)	3.480 (1.25)	3.331 (1.27)
	Casual	30 (4.1)	3.067 (1.31)	3.333 (0.99)
Specialty	Surgical	141 (19.2)	3.604 (1.13)	3.482 (1.23)
	Medical	276 (37.6)	3.598 (1.19)	3.489 (1.21)
	Critical Care	318 (43.3)	3.579 (1.17)	3.553 (1.14)
Specialized course	Yes	222 (30.2)	3.694 (1.14)	3.703 (1.09)
	No	513 (69.8)	3.546 (1.18)	3.435 (1.22)

**Table 2:** Descriptive results for continuous covariates (N = 748)

**Variables**	**Mean (SD)**	**Aggregation Statistics**				
		**ICC(1)**	**ICC(2)**	**η**^**2**^	**ω**^**2**^	**p**
Attitude towards research	4.188 (0.47)	0.027	0.387	0.068	0.026	0.017
Belief suspension (Willingness)	3.956 (0.66)	0.041	0.494	0.081	0.040	0.001
Belief suspension (Implement)	3.592 (0.86)	0.068	0.622	0.105	0.065	0.000
SF-8^TM^ (Physical health status)	50.107 (7.53)	0.018	0.296	0.060	0.018	0.066
SF-8^TM^ (Mental health status)	48.397 (8.94)	0.016	0.268	0.057	0.015	0.091
MBI Exhaustion	2.150 (1.18)	0.077	0.653	0.113	0.074	0.000
MBI Cynicism	1.739 (1.19)	0.031	0.421	0.071	0.030	0.009
MBI Efficacy	4.252 (1.00)	0.054	0.562	0.092	0.052	0.000
Adequate orientation	4.018 (0.75)	0.023	0.349	0.063	0.022	0.033
Job satisfaction	4.007 (0.77)	0.061	0.597	0.099	0.059	0.000
Problem solving	3.825 (0.36)	0.000	0.000	0.039	0.000	0.613
Research use in past	4.004 (0.72)	0.031	0.418	0.071	0.029	0.010
ACT Leadership	3.719 (0.76)	0.066	0.614	0.104	0.064	0.000
ACT Culture	3.848 (0.54)	0.101	0.718	0.137	0.098	0.000
ACT Evaluation	3.181 (0.83)	0.318	0.913	0.340	0.310	0.000
ACT Formal Interactions	1.798 (1.01)	0.144	0.793	0.176	0.140	0.000
ACT Informal Interactions	5.376 (1.66)	0.145	0.794	0.179	0.142	0.000
ACT Social Capital	3.973 (0.48)	0.052	0.556	0.091	0.051	0.000
ACT Structural and Electronic Resources	4.921 (1.78)	0.078	0.658	0.116	0.076	0.000
ACT Organizational Slack-Staff	3.138 (0.98)	0.317	0.913	0.338	0.308	0.000
ACT Organizational Slack-Space	2.980 (0.89)	0.215	0.861	0.243	0.209	0.000
ACT Organizational Slack-Time	2.999 (0.60)	0.205	0.854	0.232	0.198	0.000
Support for innovation	3.456 (0.83)	0.086	0.682	0.122	0.083	0.000
% of baccalaureate	0.711 (0.45)	N/A	N/A	N/A	N/A	N/A
# of average occupied beds	25.287 (12.60)	N/A	N/A	N/A	N/A	N/A
Average patient stay	7.600 (6.03)	N/A	N/A	N/A	N/A	N/A

**Table 3:** GEE Results for Instrumental Research Use (IRU) and Conceptual Research Use (CRU) (Independent Working Correlation Structure)

**Level**	**Variables**	**IRU**		**CRU**	
		**Estimate (SE)**	**p-value**	**Estimate (SE)**	**p-value**
**Individual level covariates (N = 748)**	Attitude towards research	0.168 (0.107)	0.118	0.035 (0.095)	0.712
	Belief suspension (Implement)	**0.159 (0.054)**	**0.004**	**0.142 (0.062)**	**0.023**
	Belief suspension (Willingness)	-0.020 (0.050)	0.687	-0.003 (0.074)	0.968
	SF-8^TM^ (Physical health status)	0.005 (0.006)	0.414	-0.002 (0.007)	0.812
	SF-8^TM^ (Mental health status)	-0.001 (0.005)	0.839	-0.001 (0.005)	0.921
	MBI Emotional Exhaustion	0.063 (0.057)	0.266	0.072 (0.054)	0.184
	MBI Cynicism	**-0.118 (0.050)**	**0.017**	-0.075 (0.047)	0.111
	MBI Efficacy	0.065 (0.036)	0.071	0.058 (0.043)	0.174
	Adequate orientation	0.117 (0.066)	0.074	0.047 (0.063)	0.455
	Job satisfaction	0.069 (0.067)	0.305	0.152 (0.078)	0.050
	Age^1^	-0.023 (0.025)	0.347	0.018 (0.023)	0.442
	Sex^2^	-0.152 (0.193)	0.431	-0.232 (0.225)	0.302
	Highest education - Diploma/Certificate^3^	-0.032 (0.219)	0.882	0.074 (0.235)	0.752
	Bachelor degree	0.376 (0.223)	0.091	0.287 (0.233)	0.219
	Employment status - Full time^4^	0.290 (0.275)	0.291	0.137 (0.245)	0.576
	Part time	0.294 (0.267)	0.272	0.060 (0.265)	0.820
	Problem solving	0.097 (0.121)	0.424	**0.290 (0.140)**	**0.039**
	Specialized course (Yes/No)^5^	0.084 (0.095)	0.381	0.084 (0.072)	0.245
	Research use in past	**0.221 (0.095)**	**0.020**	**0.248 (0.089)**	**0.006**
**Unit level covariates (N = 32)**	ACT Leadership	0.233 (0.144)	0.106	**0.437 (0.108)**	**<.0001**
	ACT Culture	**0.834 (0.409)**	**0.042**	**-0.654 (0.329)**	**0.047**
	ACT Evaluation	0.151 (0.124)	0.223	**0.353 (0.074)**	**<.0001**
	ACT Formal Interactions	-0.053 (0.133)	0.691	**-0.342 (0.084)**	**<.0001**
	ACT Informal Interactions	0.245 (0.137)	0.074	**0.286 (0.085)**	**0.001**
	ACT Social Capital	-0.076 (0.380)	0.841	-0.497 (0.306)	0.105
	ACT Structural and Electronic Resources	-0.280 (0.146)	0.056	0.005 (0.104)	0.964
	ACT Organizational Slack-Staff	-0.060 (0.118)	0.612	0.027 (0.070)	0.702
	ACT Organizational Slack-Space	-0.074 (0.128)	0.562	**0.247 (0.103)**	**0.016**
	ACT Organizational Slack-Time	0.121 (0.302)	0.690	-0.113 (0.228)	0.620
	Support for innovation	-0.518 (0.273)	0.057	-0.244 (0.220)	0.268
	Specialty - Critical care^6^	0.174 (0.172)	0.313	0.175 (0.146)	0.230
	Medical care	-0.032 (0.113)	0.776	**0.211 (0.093)**	**0.023**
	Average (mean) number of occupied beds	0.005 (0.005)	0.246	0.002 (0.003)	0.461
	Percentage of baccalaureate nurses	**0.629 (0.294)**	**0.032**	0.036 (0.263)	0.890
	Average patient stay	-0.026 (0.013)	0.050	-0.002 (0.010)	0.828
**QIC &working correlation**		$\hat{\rho }=0.00$		$\hat{\rho }=0.00$	
		QIC = 723.57		QIC = 704.93	

*Bold* statistically significant (p < .05).

^1^Used as continuous to examine the linear trend.

^2^Reference group = Female.

^3^Reference group = Master or higher.

^4^Reference group = Casual.

^5^Reference group = No.

^6^Reference group = Surgical care unit.

From the unit profile survey we obtained the following two variables (each assigned as unit-level measurement in our models): (1) average number of beds occupied and (2) average length of patient stay (in days). The remaining variables were obtained from the staff survey. Unit-level variables included: context (n = 10 variables, as measured by the Alberta Context Tool), support for innovation, and percentage of nurses on the unit with a baccalaureate or higher degree.

##### Context

Context was measured using the Alberta Context Tool (ACT) [33], premised on the Promoting Action on Research Implementation in Health Services framework which argues that successful implementation of research is a function of optimal levels of context, facilitation and evidence [34,35]. The Pediatric Nurse version of the ACT contained in the staff survey contains 58 items, which reflect 10 organizational context concepts: leadership, culture, evaluation, social capital, informal interactions, formal interactions, resources, and organizational slack (staff, space, and time). Scores on the ACT are obtained from individuals (in this case, nurses) and aggregated to generate unit scores for each of the 10 dimensions of the measure. Reliability and validity of scores with nurses was demonstrated previously using Time 1 data from this study [33]. In that analysis, a principal components analysis indicated a 13-factor solution. Bivariate associations between IRU (which the ACT was developed to predict) and the majority of ACT factors were statistically significant supporting construct validity. Adequate internal consistency reliability was also reported [33]. Reliability coefficients using Time 2 data (used in this paper) is also available as part of the instrument table in Additional file 2.

##### Individual variables

Demographic variables included were: age, sex, highest education, and employment status. Individual variables included were: attitude towards research, belief suspension-implement, belief suspension-willingness, physical health status, mental health status, three dimensions of burnout (exhaustion, cynicism, and efficacy), adequate orientation, job satisfaction, problem solving, attendance at specialized courses, and research use in the past.

### Analytic strategy

#### Reliability and validity of aggregated data at the unit level

Contextual concepts were hypothesized as unit-level concepts (i.e., scores on these variables were believed to be similar among nurses in a unit). While direct measurement of these concepts would be preferable, it is not for the most part possible and if done is prohibitively resource intensive (e.g., informal interactions). Therefore, in order to include unit-level estimates of organizational context dimensions in our models, we needed to obtain data from individual nurses and aggregate these data to the unit-level. Hence, the first step in our analysis was to examine the reliability and validity of the explanatory variables that we planned to aggregate to the unit-level. We calculated four standard empirical aggregation indices for this assessment: intraclass correlation 1, ICC(1) (a measure of individual score variability about the subgroup mean); intraclass correlation 2, ICC(2) (a measure of stability of aggregated data at the group level;); eta-squared, η^2^ (the proportion of variance in the individual variable); and omega-squared, ω^2^ (measures the relative strength of aggregated data as an explanatory variable).

The accepted standards for aggregation are ICC(1) values of 0.10 or greater and ICC(2) values of 0.60 or greater [36,37]. One-way ANOVA with random effects was performed on explanatory variables collected from nurses using ‘unit’ as the grouping variable. The ANOVA table was then used to calculate the aggregation indices (Table 2). We have reported the calculation and interpretation of these indices previously [26,38].

#### Modeling approach

The data collected for this study has a natural hierarchical or clustered structure, meaning nurses were nested within units, which were nested with pediatric hospitals. Each unit constitutes a somewhat unique work context that is shared by the nurses within that unit, and hence nurse responses within a unit may be correlated. There are several approaches to assessing clustered data. In this paper, we used Generalized Estimating Equations (GEE) because this provides the ability to model similarities between nurses that arise from: similarities in measured individual nurse characteristics, similarities resulting from measured unit-level characteristics, and similarities potentially arising from unmeasured unit-level characteristics via what is known as the “working correlation” [39,40]. We consider both exchangeable and independent working correlation structures because these comprise the most reasonable alternatives in the context of data clustered for non-longitudinal reasons. The "exchangeable" working correlation estimates the degree of coordination on the dependent variable (over and above the coordination between nurses resulting from the other modeled variables) between all pairs of nurses within a unit, with the same correlation being applied to all pairs of nurses within all units. It estimates the residual degree of similarity between nurses within units where the sources of that similarity remains unknown. The “independence” GEE model sets the working correlation to zero so there is no residual or beyond-model similarity between nurses within units. This is known as a marginal, or population averaged model [39] and results in consistent and robust estimates despite the unspecified sources of the working correlation. We also report values for Quasi-Akaike’s Information Criteria (QIC) regarding model fit. By conducting and examining the findings from both exchangeable and independent working correlation structures, we, in essence, were able to conduct a sensitivity analysis with respect to correlation structure.

## Results

### Sample characteristics

We were able to contact (and thus invite to participate) 80% of all eligible participants; the number of eligible participants was determined by the study research nurse in consultation with the unit managers while completing the unit profile survey. The overall response rate for nurses (RNs and LPNs) at Year 2 was 39% (n = 779). Missing data was minimal; all nurse cases had > 90% complete data. Therefore, we did not delete any complete nurse cases based on missing data. Further, in our analyses missing data on any item was treated as missing using listwise deletion. A total of 13 cases were deleted because they did not meet eligibility criteria (i.e., they worked on their nursing unit for less than three months). For the current analysis, we desired a homogenous sample and therefore deleted the 31 LPN cases, leaving us with an analytic sample of 735 RNs. A summary of the demographic data pertaining to the RN sample is presented in Table 1.

### Reliability and validity of aggregated data

The aggregation statistics supported aggregation to the unit level for the context survey variables theorized to be unit-level (Table 2). These variables had ICC(1) values significantly greater than 0 and several, greater than the 0.10 standard for aggregation indicating a degree of perceptual agreement among the nurses within the units about the values on these explanatory variables. The ICC(2) values were also near or exceeded the accepted standard of 0.60 for unit-level aggregation. Finally, the relative effect sizes (η^2^, ω^2^) were as expected, on average, low to moderate suggesting that as we aggregated these variables, the unit-averages were able to contribute ‘low to moderate’ portions of the overall variance in the variable.

### Results of the GEE analysis

The GEE analysis for IRU and CRU using an independent or 0.0 working-correlation structure are summarized in Table 3. Initially, we also estimated GEE models employing an exchangeable working correlation but this resulted in unreasonable (i.e., negative) working correlation estimates for both IRU and CRU. Therefore, in line with recommendations by Hanley and colleagues [41], we report the results for the independent working correlation models. The estimates from both models (exchangeable and independent working correlations) were highly similar (see Additional file 3).

#### Instrumental research utilization

Four variables were identified as significant predictors of IRU at the 5% level. Significant predictors at the individual-level were: belief suspension-implement (estimate: 0.159) and research use in the past (estimate: 0.221). Significant predictors at the unit (context) level were: culture (estimate: 0.834) and proportion of nurses possessing a baccalaureate degree or higher (estimate: 0.629) (Table 3).

#### Conceptual research utilization

Ten variables were identified as significant predictors of CRU at the 5% level. Significant predictors at the individual-level were: belief suspension-implement (estimate: 0.142), problem solving ability (estimate: 0.290), and research use in the past (estimate: 0.248). Significant predictors at the unit (context) level were: leadership (estimate: 0.437), culture (estimate: -0.654), evaluation (estimate: 0.353), formal interactions (estimate: -0.342), informal interactions (estimate: 0.286), organizational slack-space (estimate: 0.247), and unit specialty (medicine compared to surgery) (estimate: 0.211) (Table 3). Overall, only two variables were significant predictors of both IRU and CRU: belief suspension-implement (an individual-level variable) and culture (a contextual variable).

## Discussion

Our findings raise several important issues for discussion. First, several dimensions of organizational context predict pediatric nurses’ use of research findings in clinical practice. Second, there are several differences with respect to which dimensions of context predict IRU compared to CRU. Third, select individual (nurse) characteristics remain significant predictors of nurses’ use of research after controlling for organizational context.

### The importance of organizational context

Our findings show that certain dimensions of context predict research use by pediatric nurses. This builds on our previous work in which we reported that pediatric nursing units where nurses reported the highest mean overall research utilization scores (defined as any kind of research use) clustered together on the following contextual factors: unit culture (measured by work creativity, work efficiency, questioning behavior, co-worker support, and the importance nurses place on access to continuing education) and environmental complexity (measured by changing patient acuity and re-sequencing of work) [42]. In a subsequent study, we explored the importance of three dimensions of context (culture, leadership, and evaluation using the measures used in the current study) in relation to instrumental and conceptual research use [26]. In that study, we reported that pediatric nursing units with the highest IRU and CRU scores by nurses had a more positive context (i.e., nurses perceived the culture, leadership, and evaluation of the unit to be positive) compared to units where nurses reported lower research use scores [26]. In our current study, we extended these findings by showing that not only do these dimensions cluster around research use but also that they *and additional dimensions of context* (i.e., formal interactions, informal interactions, and organizational slack) are important predictors of nurses’ instrumental and/or conceptual research use.

In recent years, we have seen a number of studies examining the organizational ‘social’ context in pediatric social services [4,5,13,43,44]. In this work, Glisson and colleagues reported statistically significant associations between organizational climate (defined as ‘the way people perceive their work environment’ [13]) and child health outcomes (e.g., child psychological functioning) [13]. They also reported that child mental health and social service organizations have a variety of culture profiles and that those profiles are associated with criteria that are important to research implementation [5,13,44]. Most recently, Aarons and colleagues [45] conducted a national survey with 1,112 mental health service providers in 100 mental health service (including pediatric) institutions in 26 US states, and found that more proficient organizational cultures and more engaged and less stressful organizational climates were associated with positive clinician attitudes toward adopting evidence-based practice generally. These findings, although conducted in pediatric social services and mental health, align with the findings we report in this paper. Further, our findings also offer the first empirical support of several additional dimensions of context relating to communication (i.e., formal interactions, informal interactions, and organizational slack-space) not previously reported that show promise as important to research use by pediatric healthcare professionals.

### Context and CRU

The contextual dimensions that predicted IRU and CRU differed substantially with only one dimension (culture) displaying significance with both IRU and CRU. The remaining contextual dimensions predicted either IRU or CRU, and substantially more contextual dimensions predicted CRU than IRU. There are several plausible reasons for this finding. First, while not necessary for IRU, CRU *may* be a precursor to IRU in some instances. That is, using research to change one’s own thinking, while not necessary may in some cases, lead to using research to change one’s behavior. Second, changing the behavior of healthcare professionals (IRU) is both a difficult and multi-component process. It involves consideration of multiple contextual, personal, and behavioral factors. The Theoretical Domains Framework [46,47], for instance identifies 128 constructs tapping different factors important to health professional behavior change. It is therefore plausible that more and different factors than those measured in our study contribute to IRU compared to CRU. Finally, our modeling approach only allowed us to identify which contextual dimensions predict research use and not to determine ‘how’ context influences research use. It may be that different interactions between contextual dimensions or between context and individual characteristics produce different effects on IRU and CRU or that context indirectly influences IRU through CRU. It is also possible that CRU influences the context variables, or other individual level predictor variables in our models. For example, a nurse who is high in CRU may become higher in ‘efficacy’, or view the ‘leadership’ as superior because they are high in CRU.

The majority of our findings revealed that a more positive context predicted higher conceptual research use as hypothesized; however, we did observe two unexpected findings in relation to CRU. Culture (defined as “the way that “we do things in our organizations and work units” with items generally reflecting a supportive work culture [33]) and formal interactions (defined as “formal exchanges that occur between individuals working within an organization (unit) through scheduled activities that can promote the transfer of knowledge” [33]) both displayed negative estimates with CRU indicating a more positive culture and participating in more formal interactions leads to less CRU by nurses. These findings are contrary to theories of context and research use (e.g., PARiHS [34,35]); however, these theories focus on instrumental research use. There is currently no theory and/or empirical research examining contextual predictors of CRU. Therefore, these two findings, while unexpected require replication in future studies before drawing conclusions on these relationships. If the finding persists, it should be investigated qualitatively to better understand the mechanism by which this may occur.

### The importance of individual characteristics

Consistent with a recent systematic review [48], our findings point to the continued importance of individual characteristics. Two individual characteristics in particular, *belief suspension* and *use of research in the past*, were significant predictors of both IRU and CRU in our models.

#### Belief suspension

The Theory of Planned Behavior (TPB) [49] proposes that motivation determines behavior, and therefore the best predictors of behavior are factors that predict or determine motivation. The theory further asserts that motivation strength is determined by three variables: attitudes, subjective norms, and perceived behavioral control, which in turn are based upon salient *beliefs* about the behavior [49]. Belief suspension in our study refers to this - an individual’s perception of the degree to which they are able to suspend such beliefs in order to use research. Godin and colleagues [50], in a recent systematic review, found healthcare professionals’ beliefs about their own capabilities and the consequences of their behavior to be consistently and positively associated, at statistically significant levels, with their motivation to change their behavior. This is in line with our findings that belief suspension is important to nurses’ IRU. Future research should focus on determining how belief suspension leads to research use, both instrumentally and conceptually.

#### Research use in the past

‘Research use in the past’ was operationalized as the use of research findings to change practice in the **past** (i.e. greater than six months ago). Action theories such as Operant Learning Theory (OLT), which are also frequently used to explain behavior, postulate that past behavior is one of the most predictive factors for future behavior. According to OLT, as rewarded behaviours are repeated, they can become ‘habitual’ in the context in which they are rewarded. As a result, the frequency of past behavior can be a powerful predictor of future behavior [51,52]. Our findings support this with respect to both IRU and CRU. Future research that explores the relationship between past behavior and research use however is needed.

### Limitations

First, our sample is drawn from academically affiliated hospitals only and only includes nurses from pediatric medical, surgical, and intensive care units. Since we were able to contact (to invite to participate) 80% of all eligible participants, we believe our sample is representative of Canadian pediatric nurses in the medical, surgical, and intensive care units that we surveyed. However, our results should not be generalized beyond these settings or this particular group of nurses. For example, we do not know if the findings would be similar for nurses from community geriatric settings. Second, it is important to note that we did not collect data on several potentially important contextual factors such as overall hospital size, functional differentiation, decision-making structure, data infrastructure, and information systems [17,18]; future research should incorporate these systems-level dimensions. Third, as with all modeling techniques, our approach is not without limitation. While the GEE model is an appropriate approach for our data, allowing us to reduce the potential for biased estimates by accounting for the naturally nested structure in our data, it is possible that the proportion of the intra-group variability may change with some explanatory variables, which our GEE models do not allow for. An alternate model such as a mixed effect model with heteroscedasticity would account for such changes in covariance and could be potentially done in the future with larger datasets and fewer explanatory variables (as theory on what dimensions of context predict research use advances, we will be able to narrow the number of explanatory variables to allow such testing).

## Conclusions

Several dimensions of organizational context, as well as individual characteristics, were shown to be important predictors of research use. Interestingly, different dimensions of context predicted IRU and CRU and substantially more dimensions predicted CRU compared to IRU. Unit culture was shown to be a significant predictor of both kinds of research use and formal and informal communications between nurses and other professionals was shown to be important to nurses’ CRU. Future research is now needed to understand how these dimensions of context work to influence research use. This study offers empirical evidence of the importance of context to research use by identifying several modifiable dimensions of context that predict IRU and CRU. These findings will be used in our future research to tailor our interventions to the local context with the aim of improving research use by nurses to achieve better patient and eventually, system, outcomes.

## Competing interests

The authors declare that they have no competing interests.

## Authors’ contributions

JES, CAE, SS, GGC and BS participated in designing the study and securing its funding. JES, CAE, LH and SHK designed the analytic plan for the analyses presented in this paper. SHK conducted the statistical analysis; JES, CAE, LH, and SHK interpreted the statistical analysis. JES, SDS and GGC participated in drafting the manuscript. All authors provided critical commentary on the manuscript and approved the final version.

## Pre-publication history

The pre-publication history for this paper can be accessed here:

http://www.biomedcentral.com/1472-6963/13/351/prepub

## Supplementary Material

Additional file 1 Staff Inclusion and exclusion criteria. Click here for file

Additional file 2 Description of independent variables. Click here for file

Additional file 3 GEE Results for independent and exchange working correlation structures. Click here for file
