# Supplementary material for: The influence of organizational context on the use of research by nurses in Canadian pediatric hospitals
Source: BMC Health Serv Res. 2013 Sep 14;13:351. doi: 10.1186/1472-6963-13-351 (PMC3848566; doi:10.1186/1472-6963-13-351)
Supplement: Additional file 2 — Description of independent variables. [file 1472-6963-13-351-S2.docx]

| **Level of Data** | **Variable** | **Definition** | **Source** | **How Measured** | **How Final Score Derived** | **Alpha** |
| --- | --- | --- | --- | --- | --- | --- |
| Individual | Attitude towards research | An individual’s perception of their attitude towards research knowledge expressed along a continuum of negative to positive | TROPIC  Survey | 6 items scored on a 5-point likert agreement scale (Strongly Disagree to Strongly Agree). | An overall attitude towards research score is derived by taking the mean of the 6 items | 0.768 |
|  | Belief suspension (Implement) | An individual’s perception of the degree to which they are able to suspend previously held beliefs in order to implement a research based change. | TROPIC  Survey | 3 items scored on a 5-point likert frequency scale (Never to Almost Always). | An overall belief suspension score is derived by taking the mean of the 3 items | 0.842 |
|  | Belief suspension (Willingness) | An individual’s perception of the degree to which they are able to suspend previously held beliefs in order to be willing to implement a research based change. | TROPIC  Survey | 3 items scored on a 5-point likert agreement scale (Strongly Disagree to Strongly Agree). | An overall belief willingness score is derived by taking the mean of the 3 items | 0.784 |
|  | SF-8TM  (Physical health status) | An individual’s perception of the status of their physical and mental health over the past 4 weeks | TROPIC  Survey | 8 items scored on 5 or 6 point scales depending on the item | Scoring is done using a proprietary algorithm obtained when permission to use the scale is granted to produce a summary mental and physical health score (0-100%) | N/A |
|  | SF-8TM  (Mental health status) |  |  |  |  | N/A |
|  | MBI Emotional Exhaustion | An individual’s perception of their level of burnout. Burnout refers to a debilitating psychological condition brought about by unrelieved work stress (Maslach, 1982). Three subscales are measured: exhaustion, cynicism, efficacy | TROPIC  Survey | 3 items scored on a 7-point likert frequency scale (Never to Daily) for each subscale | The overall score for each subscale is derived by taking the mean of the 3 items. | 0.756 |
|  | MBI Cynicism |  |  |  |  | 0.704 |
|  | MBI Efficacy |  |  |  |  | 0.642 |
|  | Adequate orientation | An individual’s perception of whether they have had enough orientation to carry out their job effectively and safely. | TROPIC  Survey | A single item scored on a 5-point likert agreement scale (Strongly Disagree to Strongly Agree) | An overall adequate orientation score is same as the original item score on 5-point likert scale | N/A |
|  | Job satisfaction | An individual’s perception of whether they are “satisfied” in their job (e.g. satisfied being a healthcare aide in long term care) | TROPIC  Survey | A single item scored on a 5-point likert agreement scale (Strongly Disagree to Strongly Agree) | An overall job satisfaction score is same as the original item score on 5-point likert scale | N/A |
|  | Age | An individual’s age | TROPIC  Survey | Asked to indicate age according to category (e.g., < 20 years, 20-24 years….65-70 years, >70 years) | Recode to 10 year categories (20-29 years, 30-39 years etc.) | N/A |
|  | Sex | An individual’s sex | TROPIC  Survey | Asked for their sex:  male or female | - Male  - Female (ref) | N/A |
|  | Highest education | Highest level of education obtained | TROPIC  Survey | Asked if completed Diploma/Certificate (was selected/not selected) and if completed Bachelor degree (was selected/not selected) and if completed Master degree or higher (was selected/not selected) | Derived as highest level:  - Diploma/Certificate  - Bachelor degree  - Master or higher (ref) | N/A |
|  | Employment status | Employment status | TROPIC  Survey | Asked for their current employment status | - Full Time  - Part Time  - Casual (ref) | N/A |
|  | Problem solving | The ability of an individual to implement behaviors that reflect a goal directed sequence of cognitive operations utilized to cope with challenges or demands^1^ | TROPIC  Survey | 10 items scored on a 5-point likert agreement scale (Strongly Disagree to Strongly Agree). | Recode the 3 negatively worded items and then take the average of the 10 items using the recoded items to get the overall score for Problem Solving. | 0.720 |
|  | Specialized course | A nurse has completed any specialized course | TROPIC  Survey | Asked if they completed specialized courses (yes/no) | - Yes  - No (ref) | N/A |
|  | Research use in past | Intent to use research in the past | TROPIC  Survey | A single item scored on a 5-point likert agreement scale (Strongly Disagree to Strongly Agree) | An overall intent to use research score is same as the original item score on 5-point likert scale | N/A |
| Unit | ACT Leadership | An individual’s perception of leadership on their unit. leadership refers to the actions of formal leaders in an organization (unit) to influence change and excellence in practice, items generally reflect emotionally intelligent leadership | TROPIC  Survey | 6 items scored on a 5-point likert agreement scale (Strongly Disagree to Strongly Agree). An overall leadership score is derived by taking the mean of the 6 items | Data collected at the individual-level were aggregated to the level of the unit by calculating group means to create unit-level scores. | 0.903 |
|  | ACT Culture | An individual’s perception of culture on their unit. Culture refers to the way that “we do things’ in our organizations and work units, items generally reflect a supportive work culture | TROPIC  Survey | 6 items scored on a 5-point likert agreement scale (Strongly Disagree to Strongly Agree). An overall culture score is derived by taking the mean of the 6 items | Data collected at the individual-level were aggregated to the level of the unit by calculating group means to create unit-level scores. | 0.746 |
|  | ACT Evaluation | An individual’s perception of evaluation on their unit. Evaluation refers to the process of using data to assess group/team performance and to achieve outcomes in organizations or units (i.e., evaluation) | TROPIC  Survey | 6 items scored on a 5-point likert agreement scale (Strongly Disagree to Strongly Agree). An overall evaluation score is derived by taking the mean of the 6 items | Data collected at the individual-level were aggregated to the level of the unit by calculating group means to create unit-level scores. | 0.912 |
|  | ACT Formal Interactions | An individual’s perception of formal interactions on their unit. Formal interactions refers to the formal exchanges that occur between individuals working within an organization (unit) through scheduled activities that can promote the transfer of knowledge | TROPIC  Survey | 4 items scored on a 5-point likert frequency scale (Never to Almost Always with a “not available” option). Recode each of the 4 item scores to ‘0’ (no interaction) - 1’ (interaction). An overall formal interactions score is derived by taking a count of the 4 items using the recoded scores. | Data collected at the individual-level were aggregated to the level of the unit by calculating group means to create unit-level scores. | 0.638 |
|  | ACT Informal Interactions | An individual’s perception of informal interactions on their unit. Informal interactions refers to the informal exchanges that occur between individuals working within an organization (unit) that can promote the transfer of knowledge | TROPIC  Survey | 9 items scored on a 5-point likert frequency scale (Never to Almost Always). Recode each of the 9 item scores to ‘0’ (no interaction) - 1’ (interaction). An overall informal interactions score is derived by taking a count of the 9 items using the recoded scores. | Data collected at the individual-level were aggregated to the level of the unit by calculating group means to create unit-level scores. | 0.816 |
|  | ACT Social Capital | An individual’s perception of social capital on their unit. Social capital refers to the stock of active connections among people. These connections are of three types: bonding, bridging, and linking | TROPIC  Survey | 6 items scored on a 5-point likert agreement scale (Strongly Disagree to Strongly Agree). An overall social capital score is derived by taking the mean of the 6 items. | Data collected at the individual-level were aggregated to the level of the unit by calculating group means to create unit-level scores. | 0.797 |
|  | ACT Structural and Electronic Resources | An individual’s perception of structural resources on their unit. Structural Electronic Resources refers to the structural elements of an organization (unit) that facilitate the ability to assess and use knowledge | TROPIC  Survey | 11 items scored on a 5-point likert frequency scale (Never to Almost Always with a ‘non accessible’ option). Recode each of the 11 item scores to ‘0’ (no resource) - 1’ (resource). An overall structural resources score is derived by taking a count of the 11 items using the recoded scores. | Data collected at the individual-level were aggregated to the level of the unit by calculating group means to create unit-level scores. | 0.723 |
|  | ACT Organizational Slack-Staff | An individual’s perception of organizational slack on their unit. Organizational slack refers to the cushion of actual or potential resources which allows an organization (unit) to adapt successfully to internal pressures for adjustments or to external pressures for changes | TROPIC  Survey | 3 items scored on a 5-point likert agreement scale (Strongly Disagree to Strongly Agree). An overall organizational slack staff score is derived by taking the mean of the 3 items | Data collected at the individual-level were aggregated to the level of the unit by calculating group means to create unit-level scores. | 0.858 |
|  | ACT Organizational Slack-Space |  | TROPIC  Survey | 4 items scored on a 5-point likert agreement scale (Strongly Disagree to Strongly Agree). An overall organizational slack time score is derived by taking the mean of the 4 items | Data collected at the individual-level were aggregated to the level of the unit by calculating group means to create unit-level scores. | 0.647 |
|  | ACT Organizational Slack-Time |  | TROPIC  Survey | 2 items scored on a 5-point likert agreement scale (Strongly Disagree to Strongly Agree). An overall organizational slack space score is derived by taking the mean of the 2 items | Data collected at the individual-level were aggregated to the level of the unit by calculating group means to create unit-level scores. | 0.784 |
|  | Support for innovation | An individual’s perception of support for new ideas on their unit | TROPIC  Survey | A single item scored on a 5-point likert agreement scale (Strongly Disagree to Strongly Agree). An overall support for innovation score is same as the original item score on 5-point likert scale | Data collected at the individual-level were aggregated to the level of the unit by calculating group means to create unit-level scores. | N/A |
|  | Specialty | Specialty of unit | TROPIC  Survey | Asked to indicate if unit was surgical, medical or critical | - Surgical care unit (ref)  - Medical care unit  - Critical care unit | N/A |
|  | Average (mean) number of occupied beds | The average number of occupied beds on their unit | TROPIC Unit Profile Form | Completed by the Unit Manager (or individual responsible for the administration of the Unit | An overall # of average occupied beds is same as the original number given on the Unit profile form survey. | N/A |
|  | Percentage of baccalaureate nurses | Average proportion of nurses who completed higher education than Bachelor degree. | TROPIC  Survey | Generate a highest education at the individual level data. | Data collected at the individual-level were aggregated to the level of the unit and then proportion of nurses who has a higher education than Bachelor degree is calculated for each unit. | N/A |
|  | Average patient stay | The average length of patient stay (in days) in the past month | TROPIC Unit Profile Form | Completed by the Unit Manager (or individual responsible for the administration of the Unit | An overall # of average occupied beds is same as the original number given on the Unit profile form survey. | N/A |

^1^ Heppner PP: *The Problem Solving Inventory (PSI): Research Manual*. Palo Alto, CA: Consulting Psychologists Press; 1988.
