# Supplementary material for: The influence of organizational context on the use of research by nurses in Canadian pediatric hospitals
Source: BMC Health Serv Res. 2013 Sep 14;13:351. doi: 10.1186/1472-6963-13-351 (PMC3848566; doi:10.1186/1472-6963-13-351)
Supplement: Additional file 3 — GEE Results for independent and exchange working correlation structures. [file 1472-6963-13-351-S3.docx]

**Dependent Variable: IRU**

| **Level** | **Variables** | **Independent** | | **Exchangeable** | |
| --- | --- | --- | --- | --- | --- |
|  |  | **Estimate (SE)** | **p-value** | **Estimate (SE)** | **p-value** |
| **Individual**  **level**  **covariates**  **(N=735)** | Attitude towards research | 0.168 (0.107) | 0.118 | 0.158 (0.108) | 0.143 |
|  | Belief suspension (Implement) | 0.159 (0.054) | 0.004 | 0.165 (0.054) | 0.002 |
|  | Belief suspension (Willingness) | -0.020 (0.050) | 0.687 | -0.022 (0.049) | 0.656 |
|  | SF-8^TM^ (Physical health status) | 0.005 (0.006) | 0.414 | 0.005 (0.006) | 0.446 |
|  | SF-8^TM^ (Mental health status) | -0.001 (0.005) | 0.839 | -0.001 (0.005) | 0.801 |
|  | MBI Emotional Exhaustion | 0.063 (0.057) | 0.266 | 0.071 (0.058) | 0.218 |
|  | MBI Cynicism | -0.118 (0.050) | 0.017 | -0.122 (0.050) | 0.014 |
|  | MBI Efficacy | 0.065 (0.036) | 0.071 | 0.068 (0.038) | 0.072 |
|  | Adequate orientation | 0.117 (0.066) | 0.074 | 0.106 (0.066) | 0.107 |
|  | Job satisfaction | 0.069 (0.067) | 0.305 | 0.061 (0.067) | 0.364 |
|  | Age^[[1]](#footnote-1)^ | -0.023 (0.025) | 0.347 | -0.028 (0.025) | 0.254 |
|  | Sex^[[2]](#footnote-2)^ | -0.152 (0.193) | 0.431 | -0.143 (0.187) | 0.444 |
|  | Highest education - Diploma/Certificate^[[3]](#footnote-3)^ | -0.032 (0.219) | 0.882 | 0.363 (0.228) | 0.111 |
|  | Bachelor degree | 0.376 (0.223) | 0.091 | -0.036 (0.224) | 0.872 |
|  | Employment status - Full time^[[4]](#footnote-4)^ | 0.290 (0.275) | 0.291 | 0.252 (0.280) | 0.368 |
|  | Part time | 0.294 (0.267) | 0.272 | 0.275 (0.270) | 0.308 |
|  | Problem solving | 0.097 (0.121) | 0.424 | 0.101 (0.122) | 0.408 |
|  | Specialized course (Yes/No) ^[[5]](#footnote-5)^ | 0.084 (0.095) | 0.381 | 0.096 (0.097) | 0.319 |
|  | Research use in past | 0.221 (0.095) | 0.020 | 0.221 (0.095) | 0.021 |
| **Unit**  **level**  **covariates**  **(N=32)** | ACT Leadership | 0.233 (0.144) | 0.106 | 0.307 (0.149) | 0.040 |
|  | ACT Culture | 0.834 (0.409) | 0.042 | 0.770 (0.457) | 0.092 |
|  | ACT Evaluation | 0.151 (0.124) | 0.223 | 0.198 (0.127) | 0.121 |
|  | ACT Formal Interactions | -0.053 (0.133) | 0.691 | -0.025 (0.157) | 0.872 |
|  | ACT Informal Interactions | 0.245 (0.137) | 0.074 | 0.143 (0.119) | 0.231 |
|  | ACT Social Capital | -0.076 (0.380) | 0.841 | 0.171 (0.374) | 0.648 |
|  | ACT Structural and Electronic Resources | -0.280 (0.146) | 0.056 | -0.134 (0.091) | 0.138 |
|  | ACT Organizational Slack-Staff | -0.060 (0.118) | 0.612 | -0.081 (0.127) | 0.525 |
|  | ACT Organizational Slack-Space | -0.074 (0.128) | 0.562 | -0.036 (0.137) | 0.793 |
|  | ACT Organizational Slack-Time | 0.121 (0.302) | 0.690 | 0.084 (0.315) | 0.791 |
|  | Support for innovation | -0.518 (0.273) | 0.057 | -0.586 (0.303) | 0.053 |
|  | Specialty - Critical care^[[6]](#footnote-6)^ | 0.174 (0.172) | 0.313 | 0.105 (0.190) | 0.580 |
|  | Medical care | -0.032 (0.113) | 0.776 | -0.032 (0.124) | 0.797 |
|  | Average number of occupied beds | 0.005 (0.005) | 0.246 | 0.003 (0.005) | 0.511 |
|  | Percentage of baccalaureate nurses | 0.629 (0.294) | 0.032 | 0.537 (0.337) | 0.112 |
|  | Average patient stay | -0.026 (0.013) | 0.050 | -0.014 (0.013) | 0.270 |
| **QIC & working correlation** | | $\hat{\rho}$=0.00  QIC=723.25 | | $\hat{\rho}$=-0.01577  QIC=712.31 | |

**Dependent Variable: CRU**

| **Level** | **Variables** | **Independent** | | **Exchangeable** | |
| --- | --- | --- | --- | --- | --- |
|  |  | **Estimate (SE)** | **p-value** | **Estimate (SE)** | **p-value** |
| **Individual**  **level**  **covariates**  **(N=735)** | Attitude towards research | 0.035 (0.095) | 0.712 | 0.034 (0.096) | 0.720 |
|  | Belief suspension (Implement) | 0.142 (0.062) | 0.023 | 0.141 (0.062) | 0.023 |
|  | Belief suspension (Willingness) | -0.003 (0.074) | 0.968 | 0.004 (0.074) | 0.956 |
|  | SF-8^TM^ (Physical health status) | -0.002 (0.007) | 0.812 | -0.002 (0.007) | 0.767 |
|  | SF-8^TM^ (Mental health status) | -0.001 (0.005) | 0.921 | -0.001 (0.005) | 0.852 |
|  | MBI Emotional Exhaustion | 0.072 (0.054) | 0.184 | 0.074 (0.054) | 0.168 |
|  | MBI Cynicism | -0.075 (0.047) | 0.111 | -0.076 (0.047) | 0.100 |
|  | MBI Efficacy | 0.058 (0.043) | 0.174 | 0.064 (0.044) | 0.144 |
|  | Adequate orientation | 0.047 (0.063) | 0.455 | 0.044 (0.064) | 0.494 |
|  | Job satisfaction | 0.152 (0.078) | 0.050 | 0.150 (0.078) | 0.054 |
|  | Age^[[7]](#footnote-7)^ | 0.018 (0.023) | 0.442 | 0.015 (0.023) | 0.505 |
|  | Sex^[[8]](#footnote-8)^ | -0.232 (0.225) | 0.302 | -0.227 (0.223) | 0.308 |
|  | Highest education - Diploma/Certificate^[[9]](#footnote-9)^ | 0.074 (0.235) | 0.752 | 0.293 (0.234) | 0.210 |
|  | Bachelor degree | 0.287 (0.233) | 0.219 | 0.089 (0.235) | 0.705 |
|  | Employment status - Full time^[[10]](#footnote-10)^ | 0.137 (0.245) | 0.576 | 0.109 (0.245) | 0.656 |
|  | Part time | 0.060 (0.265) | 0.820 | 0.036 (0.263) | 0.891 |
|  | Problem solving | 0.290 (0.140) | 0.039 | 0.281 (0.138) | 0.042 |
|  | Specialized course (Yes/No) ^[[11]](#footnote-11)^ | 0.084 (0.072) | 0.245 | 0.088 (0.071) | 0.210 |
|  | Research use in past | 0.248 (0.089) | 0.006 | 0.248 (0.088) | 0.005 |
| **Unit**  **level**  **covariates**  **(N=32)** | ACT Leadership | 0.437 (0.108) | <.0001 | 0.430 (0.127) | 0.001 |
|  | ACT Culture | -0.654 (0.329) | 0.047 | -0.784 (0.349) | 0.025 |
|  | ACT Evaluation | 0.353 (0.074) | <.0001 | 0.393 (0.083) | <.0001 |
|  | ACT Formal Interactions | -0.342 (0.084) | <.0001 | -0.331 (0.102) | 0.001 |
|  | ACT Informal Interactions | 0.286 (0.085) | 0.001 | 0.203 (0.071) | 0.004 |
|  | ACT Social Capital | -0.497 (0.306) | 0.105 | -0.437 (0.343) | 0.203 |
|  | ACT Structural and Electronic Resources | 0.005 (0.104) | 0.964 | 0.095 (0.103) | 0.359 |
|  | ACT Organizational Slack-Staff | 0.027 (0.070) | 0.702 | -0.001 (0.077) | 0.990 |
|  | ACT Organizational Slack-Space | 0.247 (0.103) | 0.016 | 0.310 (0.122) | 0.011 |
|  | ACT Organizational Slack-Time | -0.113 (0.228) | 0.620 | -0.053 (0.228) | 0.816 |
|  | Support for innovation | -0.244 (0.220) | 0.268 | -0.222 (0.253) | 0.381 |
|  | Specialty - Critical care^[[12]](#footnote-12)^ | 0.175 (0.146) | 0.230 | 0.090 (0.134) | 0.501 |
|  | Medical care | 0.211 (0.093) | 0.023 | 0.244 (0.106) | 0.022 |
|  | Average number of occupied beds | 0.002 (0.003) | 0.461 | 0.002 (0.003) | 0.512 |
|  | Percentage of baccalaureate nurses | 0.036 (0.263) | 0.890 | -0.067 (0.276) | 0.810 |
|  | Average patient stay | -0.002 (0.010) | 0.828 | 0.005 (0.009) | 0.596 |
| **QIC & working correlation** | | $\hat{\rho}$=0.00  QIC=713.25 | | $\hat{\rho}$=-0.01577  QIC=704.75 | |

1. Used as continuous to examine the linear trend [↑](#footnote-ref-1)
2. Reference group = Female [↑](#footnote-ref-2)
3. Reference group = Master or higher [↑](#footnote-ref-3)
4. Reference group = Casual [↑](#footnote-ref-4)
5. Reference group = No [↑](#footnote-ref-5)
6. Reference group = Surgical care unit [↑](#footnote-ref-6)
7. Used as continuous to examine the linear trend [↑](#footnote-ref-7)
8. Reference group = Female [↑](#footnote-ref-8)
9. Reference group = Master or higher [↑](#footnote-ref-9)
10. Reference group = Casual [↑](#footnote-ref-10)
11. Reference group = No [↑](#footnote-ref-11)
12. Reference group = Surgical care unit [↑](#footnote-ref-12)
